# One Earth + One Health: An Agile, Evolutionary, System-of-Systems Convergence Paradigm

**DOI:** 10.1021/acs.est.5c11895

**Published:** 2026-03-26

**Authors:** John C. Little, Roope O. Kaaronen, Michael Muthukrishna, Sondoss Elsawah, Max S. Bennett, Inas Khayal, Janne I. Hukkinen, C. Michael Barton, Anthony J. Jakeman, Amro M. Farid

**Affiliations:** † Department of Civil and Environmental Engineering, 1757 Virginia Tech, Blacksburg, Virginia 24061, United States; ‡ Helsinki Collegium for Advanced Studies, University of Helsinki, Helsinki 00014, Finland; § Department of Psychological and Behavioural Science, London School of Economics and Political Science, London WC2A 2AE, U.K.; ∥ School of Engineering and Information Technology, 7800 University of New South Wales, Canberra, Australian Capital Territory 2600, Australia; ⊥ Fenner School of Environment and Society, Australian National University, Canberra, Australian Capital Territory 0200, Australia; # Department of Computer Science, Columbia University, New York, New York 11238, United States; ∇ Departments of Oncology and Industrial & Systems Engineering, 2954 Wayne State University, Detroit, Michigan 48201, United States; ○ Environmental Policy Research Group, 154260 University of Helsinki, Helsinki 00014, Finland; ◆ School of Complex Adaptive Systems, 7864 Arizona State University, Tempe, Arizona 85287, United States; ¶ Department of Systems Engineering, School of Engineering Sciences, Stevens Institute of Technology, Hoboken, New Jersey 07030, United States; ⬢ Department of Psychology, New York University, New York, New York 10003, United States

**Keywords:** modular evolution, scientifically falsifiable, theoretical framework, niche construction, realms of life, systems modeling language, hetero-functional graph theory

## Abstract

Evolutionary mechanisms have enabled humans to transform Earth systems. Because the resulting Anthropocene systems are highly interdependent and dynamically evolving, often with accelerating rates of cultural and technological evolution, One Earth and One Health must be framed and addressed in a holistic fashion. An agile, evolutionary, system-of-systems, convergence paradigm, which is based on a partially quantifiable, scientifically falsifiable theoretical framework, can be used to systematically identify, decompose, characterize, and then converge a nested, evolutionary ensemble of geophysical, biophysical, sociocultural, and sociotechnical systems. The paradigm includes individual organisms (spanning plants, fungi, and animals) engaging in niche construction in a global meta-ecosystem that integrates the deep evolutionary history of all Anthropocene systems. To coherently span the vast range of scales, the paradigm is divided into a somatic realm (externally oriented with respect to individual organisms) that can be applied at global, regional, urban, and local scales, as well as a visceral realm (internally oriented with respect to individual organisms) that includes organs, cells, organelles, genes, and molecules. The paradigm requires a causally coherent evolutionary framework, cross-scale, modular, and hierarchical conceptual models (based on a common language and reconciled ontology), with agile, extensible, and scalable computational frameworks, an associated decision-support system, and an educational pedagogy.

## Graphical abstract

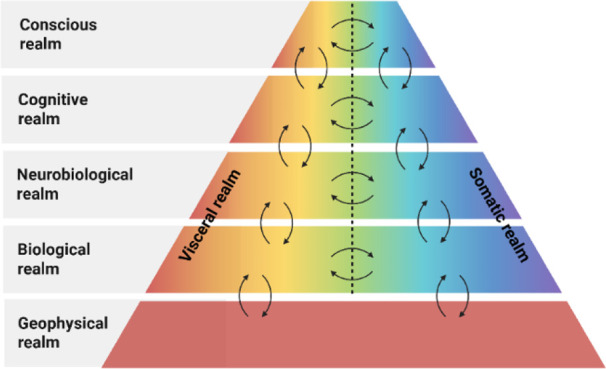

## 1 Introduction

Humans have profoundly transformed Earth’s systems, creating a broad array of deeply entwined and intractable societal challenges. For example, a recent assessment of the Planetary Boundaries framework^1^ revealed that Earth is now beyond six of nine interdependent planetary boundaries, concluding that anthropogenic impacts must be considered in a systemic context. In addition, a recent assessment of progress toward meeting the Sustainable Development Goals^2^ found no evidence that the limited environmental improvements that have been made (in forest and water ecosystems) are linked to positive social impacts. Furthermore, a recent assessment of the United Nations Framework Convention on Climate Change^3^ demonstrated that well-intentioned climate mitigation policies and measures can result in unintended consequences and problem-shifting, where efforts to curb climate change inadvertently create new environmental or socio-economic challenges. Finally, addressing climate change, emerging infectious diseases, the spread of invasive species, and food security will require a new era of continental-scale biology^4^ with multiscale, multidisciplinary theory that extends from molecules to organisms, and from ecosystems to biomes to the biosphere. Collectively, these four studies “underscore the urgent need for holistic, Earth-system-based approaches that account for system wide human-environment interactions”.^3^

The need to holistically address these interdependent societal challenges of the Anthropocene^5^ is explicitly recognized in the re-envisioned One Health approach, which aims to sustainably balance the health of humans, animals, and ecosystems.^6,7^ As shown in Figure 1, the approach intends to mobilize multiple sectors, disciplines, and communities across a range of scales and organizational levels, while simultaneously addressing the need for clean water, energy, and air, providing access to safe and nutritious food, and tackling climate change, disasters, and sustainable development.^6^ A recent assessment of the approach,^8^ which was published as part of The Lancet Series on One Health and Global Health Security,^9^ found that current frameworks do little to consider anthropogenic factors in disease, concluding that “a complex and interdependent set of challenges threaten human, animal, and ecosystem health, and that we cannot afford to overlook important contextual factors, or the determinants of these shared threats.”

**Figure 1:**
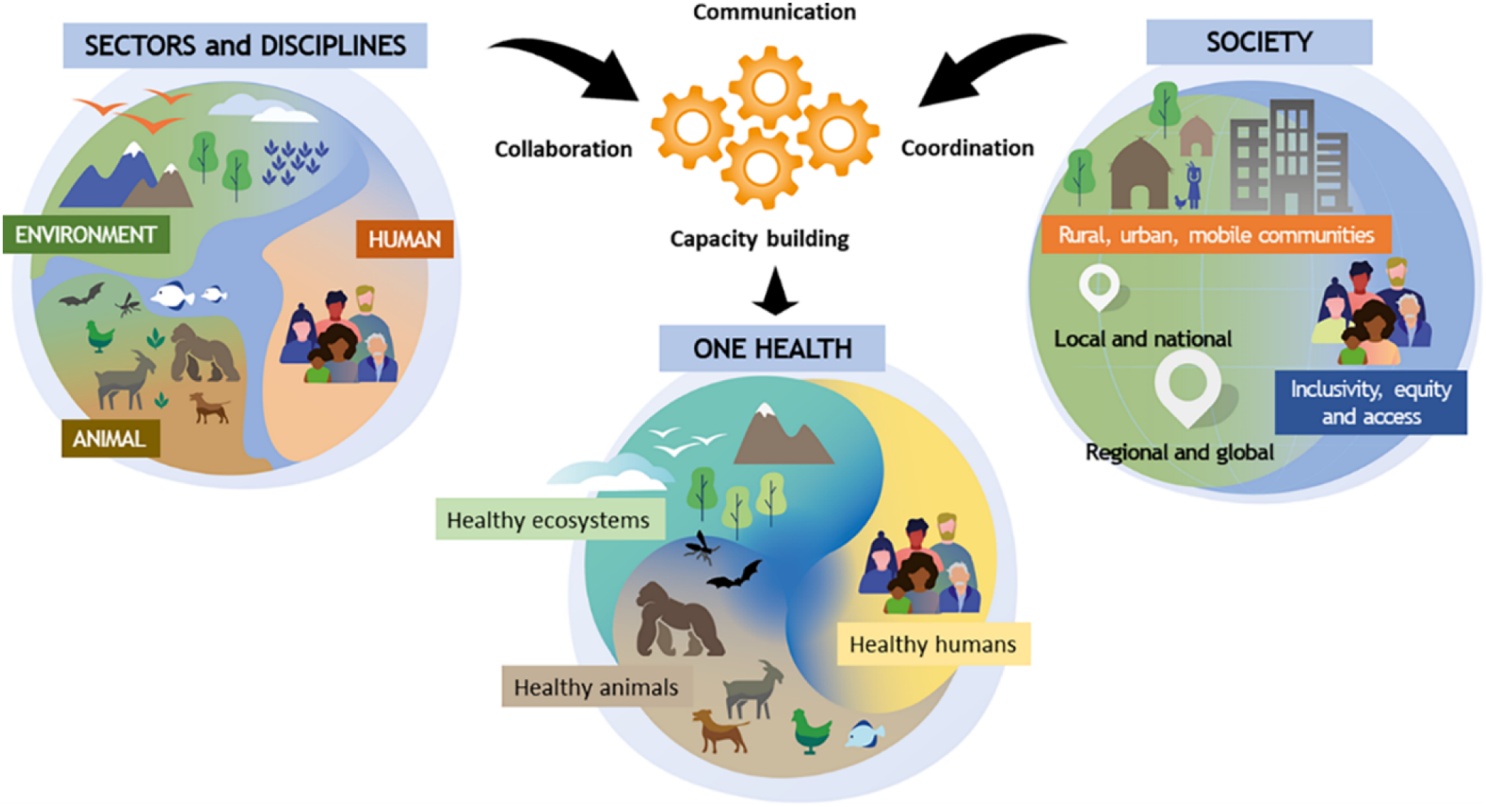
Conceptual representation of the One Health approach.^6^ To successfully address multiple globally connected and interdependent societal challenges in an integrated and holistic fashion requires extensive communication, coordination, capacity building, and collaboration.^9^ Figure reproduced from ref. 6. Available under CC-BY 1.0. Copyright 2022 PLOS Pathogens.

To address these interdependent societal challenges, we need to catalyze societal transformations with strategic interventions that can be coordinated across multiple systems and scales. However, a recent critical review^5^ argued that this is not possible with available approaches or frameworks, in agreement with five independent assessments.^1–4,8^ The critical review outlined the evolutionary mechanisms that enabled humans to transform Earth systems, culminating in the current, globally connected system of Anthropocene systems (noting that the Anthropocene is more than a time interval^10^). Because Anthropocene systems are highly interdependent and dynamically evolving, often with accelerating rates of cultural and technological evolution,^11^ the ensuing societal challenges are also highly interdependent, as is increasingly being recognized,^1,4,8,12–22^ and need to be holistically framed and addressed.^1,4–6,12,19,20,22–27^

An evolutionary perspective can also be used to gain valuable insights into earlier societal transformations, beginning with four proposed transitions in the coevolution of early humans (see Table 4 in ref. 5), and continuing with agriculture, urbanization, industrialization, and computerization. Understanding these earlier societal transformations, which enabled the coevolution of the Anthropocene and the emergence of the ensuing societal challenges, should prove valuable as we attempt to coordinate new systemic interventions.

In addition to these complex challenges, the partition of knowledge into many disciplines and subdisciplines is simultaneously one of the greatest scientific and societal challenges of our time,^5,28^ severely impeding progress because “we cannot see the forest for the trees.” The deep integration of knowledge, methods, and expertise across multiple disciplines requires convergence.^29–31^ Although the definition of convergence has evolved, it has recently been emphasized^31^ that “new frameworks, paradigms, or even disciplines can emerge from convergence research, as research communities adopt common frameworks and a new scientific language.” Convergence, therefore, facilitates transdisciplinary research,^32^ which is seen as the pinnacle of integration across disciplines.^31,33^ Because the Anthropocene systems span a vast number of disciplinary boundaries, convergence is required to address the resulting societal challenges.

In this review, we build on the previously proposed evolutionary (evo) system-of-systems (SoS) convergence paradigm,^5^ which is based on a partially quantifiable, scientifically falsifiable theoretical framework and can be used to systematically identify, decompose, characterize, and then converge a nested, evolutionary ensemble of geophysical, biophysical, sociocultural, and sociotechnical systems. The previous critical review outlined the evolutionary mechanisms that enabled humans to transform Earth systems into Anthropocene systems,^5^ but in this new review, we broaden the scope to include the coevolution of ecosystems, animals, and humans, as required for the One Health approach,^6^ and because an equivalent One Earth approach is needed^4,5,17,34^ with an evolutionary framework that integrates geophysical, biophysical, sociocultural, and sociotechnical systems at the planetary scale.^5,34^

As we will argue, an evolutionary perspective is essential because we need to understand the causally coherent, cross-scale, evolutionary mechanisms that enabled the family of societal challenges to emerge. A system-of-systems perspective is equally essential because we need to manage the unprecedented range of scale and complexity as effectively as possible. The extended evoSoS convergence paradigm will enable One Earth, One Health, and the associated family of societal challenges of the Anthropocene to be framed and addressed in an integrated fashion with causally coherent strategic interventions across multiple systems and scales. However, the development and implementation of the paradigm will require a major transformation in our approach to science and engineering, with five primary elements:

1. Causally coherent, scientifically falsifiable **theoretical framework** characterizing a system of Anthropocene systems within a planetary-scale meta-ecosystem.
2. Cross-scale, modular, hierarchical, dynamic **conceptual models** of the Anthropocene systems that are based on a common language and that reconcile disciplinary ontologies.
3. Common **computational frameworks** that build directly on the conceptual models and that are agile, extensible, and scalable.
4. Coherent **decision-support system** used to interact with the conceptual models and computational frameworks, enabling effective integration of a wide range of stakeholder perspectives spanning multiple scales and organizational levels.
5. Comprehensive **educational pedagogy** to train a new generation of Anthropocene systems integrators to develop and implement the paradigm.

To justify the five required elements of the evoSoS convergence paradigm, we more explicitly address the collective limitations of several closely related fields of research in Section 2. Our review then takes an evolutionary perspective in Section 3 and a system-of-systems perspective in Section 4. In Section 5, we outline the requirements for the primary elements, which are all crucially important to facilitate communication, coordination, capacity building, and collaboration, all of which are essential for success^6^ (see Figure 1). We conclude with a brief overview of the development and implementation of the paradigm in Section 6 and a summary of research needs in Section 7. Given the vast scope, this includes an agile approach,^27,35^ taking place in iterations, each of which produces new insights and can be refined in light of those insights, enabling a low implementation risk to the first investment and a viable roadmap toward an ambitious end goal that cannot otherwise be achieved.

## 2 Limitations of Closely Related Fields of Research

Substantial progress is being made in several closely related, interdisciplinary, and transdisciplinary fields of research, including Earth system science,^36^ integrated assessment and modeling,^37^ social-ecological systems research,^38^ sociohydrology,^39^ land systems science,^40^ socioenvironmental systems modeling,^41^ multisector dynamics,^42^ disaster resilience,^43^ circular economy,^44,45^ global polycrisis,^19,20^ and convergence research.^46,47^ Unfortunately, for our purposes, they collectively exhibit three primary limitations: (1) they are not based on the evolutionary mechanisms that gave rise to the Anthropocene and the ensuing societal challenges; (2) they include elements of social and ecological systems, but these elements are seldom based on a causally coherent evolutionary framework; and (3) they do not start with a framing that is holistic enough for addressing many interconnected societal challenges that are highly interdependent and dynamically evolving.

To give a concrete example, a review of nine publications^47–55^ in a special feature on Convergent Science for Sustainable Regional Systems, which is being published by Ecology and Society – A Journal of Integrative Science for Resilience and Sustainability, reveals that none mention the evolutionary mechanisms that gave rise to the Anthropocene. Although evolutionary approaches are being considered in social-ecological systems research (e.g., see refs. 56–59 ), they do not yet provide a causally coherent evolutionary framework, meaning that interventions across multiple systems and scales cannot be effectively coordinated. Indeed, the field of social-ecological systems acknowledges these limitations, identifying^60^ “persistent challenges, including conceptual and methodological fragmentation, difficulty in scaling localized insights to global frameworks (and vice versa), and capturing cross-scale connections and processes while retaining contextual relevance.”

Societal challenges of the Anthropocene, including One Earth and One Health, are usually addressed as if they are disconnected.^5,26,27^ As a result, many research initiatives (perhaps tens of thousands) in the closely related fields of research mentioned above are currently in progress worldwide. Many new frameworks and approaches for the various societal challenges are being produced, most involving many of the same systems (e.g., land use, watershed, energy, transportation, climate, communication, economic, and most other sociocultural systems are common across all challenges), and most will require extensive interventions within many of the same systems. The initiatives have their preferred languages, ontologies, and computational frameworks (see Section 4), with an increasing number including elements of social systems.

It is clear that urban areas drive environmental change at multiple scales^61^ and concentrate complex, multisectoral interactions within the human-Earth system.^62^ Now imagine a city within a region that has multiple interdependent societal challenges and multiple systems that are nested, highly interdependent, and dynamically evolving with accelerating rates of cultural and technological evolution. If different groups are addressing different societal challenges in the same urban area using different languages, ontologies, and computational frameworks, we have to ask:

•Can the coevolution of a system of Anthropocene systems be represented in a causally coherent fashion?

•Can the many different approaches to human behavior (e.g., see ref. 63) in sociocultural and sociotechnical systems be coherently integrated with other Anthropocene systems (e.g., geophysical and biophysical systems)?

•Can the different ontologies in multiple Anthropocene systems be reconciled?

•Can the vast complexity and deep uncertainty be simultaneously managed?

•Can the required cross-scale interventions (e.g., at local, urban, and regional scales) in multiple heterogeneous Anthropocene systems be coordinated and integrated?

So far, we are only imagining one city in one region, but there are thousands of urban areas (perhaps 10,000 cities worldwide, with about 40 megacities where the population is greater than 10 million) where similar questions apply. Again, we have to ask:

•Is the required communication, coordination, capacity building, and collaboration on multiple interdependent societal challenges at the urban scale even possible?

•Can new knowledge acquired in one urban area be rapidly included in the computational frameworks that are being developed or applied in many other urban areas?

•Can urban, regional, and global capacity-building initiatives take advantage of a common language, ontology, and computational framework?

•Are research organizations and professional societies coordinating their activities to try and change the prevailing culture in the relevant knowledge domains and disciplines?

•Are funding agencies, which typically address the range of societal challenges, coordinating their research solicitations to make the most of their limited resources?

Unfortunately, there are few positive answers to any of the ten preceding questions.

While the closely related fields of research (i.e., refs. 19,20,34,36–47 ) should be recognized for the valuable progress they are making while addressing interdisciplinary problems involving coupled systems, they do not start with a framing that is holistic enough to simultaneously address One Earth, One Health, and the associated interdependent societal challenges of the Anthropocene with causally coherent strategic interventions across multiple systems and scales.

## 3 An Evolutionary Perspective

Dynamic evolutionary mechanisms enabled billions of humans to profoundly transform Earth’s systems,^36^ creating a globally connected^1,23,34^ meta-ecosystem,^64,65^ which can be represented as a system of Anthropocene systems.^5^

### 3.1 Evolution Broadly Conceptualized

The origin story of life on Earth^66,67^ is a consequence of geological, genetic, cultural, and technological evolution,^5^ recognizing that evolution, more broadly conceptualized, is not limited to biology^68^ and requires only variation and selective retention.^69^ The Earth can be understood as an evolving planetary system^70^ with chemical elements that evolved in stars^71^ enabling the evolution of minerals^72^ on Earth, which in turn influence our globally connected meta-ecosystem and the coevolving ecological niche of life on Earth.^73,74^ Similarly, human organizations,^75^ technology,^76^ and knowledge^77^ evolve, including our knowledge^36^ of the form, function, and resulting behavior of Anthropocene systems. Indeed, our understanding of evolutionary mechanisms is also evolving.^74,78–84^

Starting with this broad evolutionary perspective, a nested evolutionary ensemble of Anthropocene systems can be identified^5^ as follows (see Figure 2): (1) Geophysical systems, which include, for example, geological, oceanic, atmospheric, climatic, and hydrological systems; (2) Biophysical systems, which integrate biological and geophysical systems and include, for example, ecological and soil systems; (3) Sociocultural systems, which are a specialized form of biophysical systems that emphasize social knowledge and culture and include, for example, cognitive, communication, education, economic, legal, and governance systems; and (4) Sociotechnical systems, which are a specialized form of sociocultural systems that emphasize technical knowledge and technology and include, for example, land-use, energy, agricultural, mining, transportation, industrial, and other infrastructure systems.

**Figure 2:**
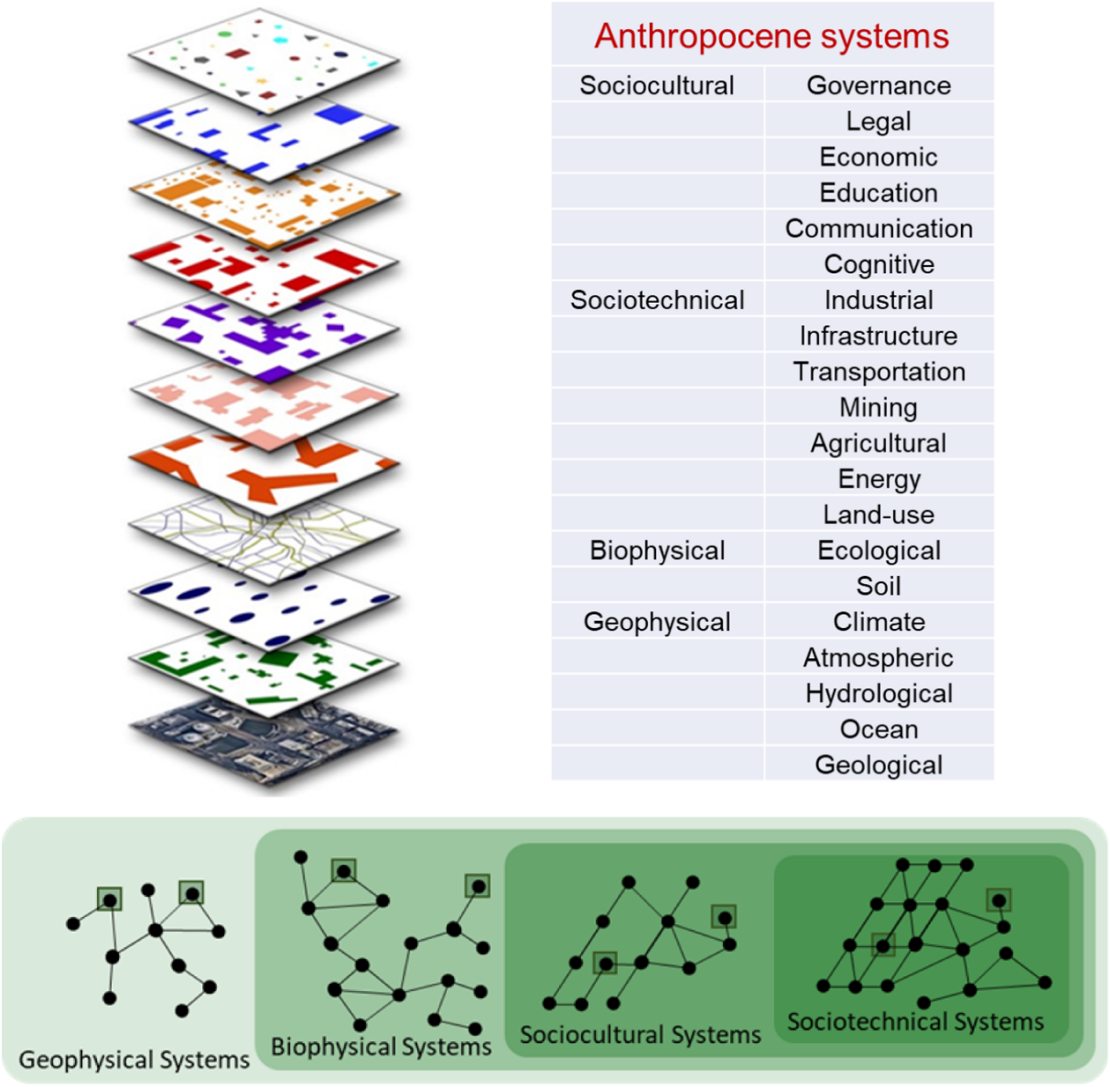
An initial list of 20 primary Anthropocene systems is shown on the right. The image on the left provides a visual representation of a system of Anthropocene systems in an urban area, with the “real world” on the bottom and 10 interdependent systems layered above. The image on the bottom illustrates the primary Anthropocene systems within a coevolving, nested, evolutionary ensemble of geophysical, biophysical, sociocultural, and sociotechnical systems.

Although cultural and technological evolution are inextricably entwined, it is nevertheless useful to distinguish between sociocultural and sociotechnical systems because technology and socially mediated technical knowledge greatly enhance human influence and accelerate coevolutionary mechanisms in the ensemble of Anthropocene systems. Earlier geophysical and biophysical systems were, of course, always connected through global climate and plate tectonics, but the more recent sociocultural and sociotechnical systems have vastly accelerated the temporal rates of interaction among the systems and vastly increased the spatial extent of interactions across the systems, creating a much more dynamic, globally connected system of Anthropocene systems.^5^

A causally coherent (i.e., mechanistically consistent) theoretical framework based on scientifically falsifiable evolutionary principles will allow researchers to derive specific predictions from more general premises, an especially urgent need in behavioral science.^85,86^ For example, a more dynamic understanding of human behavior coevolving with both biophysical and sociocultural contexts^87^ enables a better understanding of the dynamics of the Anthropocene. Without a scientifically falsifiable theoretical framework, results are neither expected nor unexpected based on how they fit into theory and cannot be related to research in other knowledge domains.^85^

Understanding coevolution more broadly, as opposed to simpler scenarios in which organisms adapt independently to a specific environment, reveals a spectrum of interactions (e.g., mutualistic, commensal, competitive, and antagonistic) that provide context and nuance to ecological strategies.^88^ In addition, organisms may actively modify their own and each other’s ecological niche, with evolution by niche construction becoming possible when these modifications influence evolutionary selection.^89^ Coevolving geophysical systems play an important role in niche construction, as organisms alter their prevailing environment and need to be included to more completely represent the coevolutionary niche. Anthropogenic change provides a compelling example of humans both intentionally and unintentionally influencing the ecological niche^73^ of life on Earth.

The evoSoS paradigm aims to coherently integrate geophysical sciences, biological sciences, health sciences, social sciences, engineering, and the humanities, providing a partially quantifiable, causally coherent, and scientifically falsifiable theoretical framework. Crucially, this framework can represent human behavior in sociocultural systems.^85^ Unfortunately, the interdependent relationship between human behavior and context has largely been ignored,^90^ although progress has been made by environmental and ecological psychology^83,91^ as well as historical psychology.^92^ Building on these and similar initiatives,^93^ the evoSoS convergence paradigm enables the integration of behavioral science – including the physical, social, and evolutionary contexts that shape perception, deliberation, and inferential reasoning – with the geophysical, biophysical, sociocultural, and sociotechnical context in which the behavior occurs.

Human behavior^92^ is shaped by billions of years of genetic evolution, millions of years of cultural evolution, and a short lifetime of accumulated knowledge, offering levers for behavioral change.^94^ Several major evolutionary mechanisms (e.g., kinship, reciprocity, status, leaders, signaling, punishment, emotions, rituals, norms, and institutions)^69,95–98^ can be used to explain human cooperation and competition in sociocultural systems. There are other mechanisms and elements of sociocultural systems that can be considered (e.g., see Table 4 and the Appendix in ref. 5), but as an illustrative starting point, a generic model of a sociocultural system might be represented^5^ as follows. Individuals in sociocultural systems process information using their own cognitive systems, cooperate and compete with other individuals using communication systems, acquire and lose status and leadership positions, acquire and forget knowledge, norms, and institutions, and form alliances with other individuals. Similarly, groups of individuals cooperate and compete with other groups using communication systems, acquire and lose status, acquire and forget knowledge, norms, and institutions, and form alliances with other groups. Governance, legal, economic, and educational systems guide and constrain the coevolving dynamics.

The resulting social dynamics involve individuals, groups, and groups of groups, with overlapping versions of these modular, scalable, agent-based structures (e.g., see ref. 99) propagating through all sociocultural systems. For example,^5^ cultural variants (e.g., skills, tools, habits, customs, rituals, norms, and institutions) can be learned or acquired socially.^83,100^ Cultural transmission occurs when a cultural variant is learned with sufficiently few errors such that even small, unobvious improvements are retained, and cultural evolution occurs when small improvements to existing cultural variants spread through populations.^100^

Once it is understood that humans evolved from unicellular organisms through cooperation, codependence, collaboration, and competition, and that this is also the case for plants, fungi, and animals, the interrelatedness of all species on Earth can be embraced,^81,83,101^ with their evolved modularity providing great potential for improving our understanding of the interconnected nature of Anthropocene systems^5^ (see Figure 3). Indeed, the coevolutionary ecological strategies already mentioned (mutualistic, commensal, competitive, and antagonistic)^88^ are essentially the same as the coevolutionary human strategies (cooperation, codependence, collaboration, and competition). Furthermore, these ecological and human strategies are essentially equivalent to archetypal cellular strategies, providing persuasive evidence for the cell as the mechanistic basis for the evolution of life.^74,102,103^

**Figure 3:**
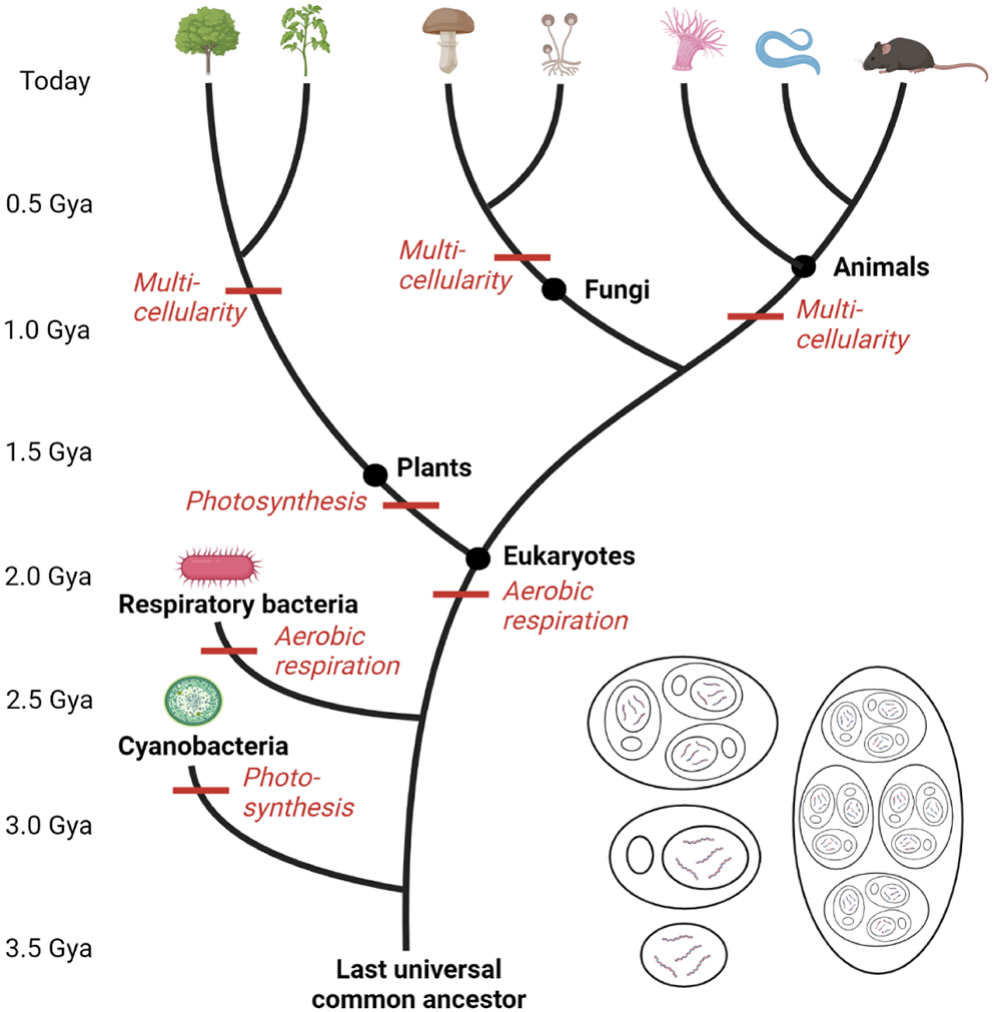
Evolutionary tree of life, modified from,^104^ with time shown in units of billions of years ago (Gya). The four images on the bottom right provide a simplified representation, modified from,^105^ of the modular nested evolutionary hierarchy for individual organisms, with a unicellular prokaryote, a unicellular eukaryote, a simple multicellular eukaryote, and a complex multicellular eukaryote.

### 3.2 The Realms of Life on Earth

Human initiatives to address societal challenges of the Anthropocene will require coordinated strategic interventions^106^ across multiple systems and scales,^107^ but a more holistic framing is needed. The best way to understand a system of coevolved Anthropocene systems is to characterize the evolutionary mechanisms that caused their form, function, and resulting behavior to evolve. Although developed while focusing on human consciousness, LeDoux’s four realms of existence^105^ provide a coherent evolutionary context for our approach to these challenges and can be summarized as follows: The biological realm spans all biology, including plants, fungi, and animals, as shown in Figure 3. The neurobiological realm is facilitated by nervous systems, which evolved in all animals, enabling control of their bodies with speed and precision that is not possible in other forms of life. Some animals with nervous systems have a cognitive realm, enabling the use of mental models to control a wide range of behaviors. Finally, the conscious realm enables inner experiences of, and thoughts about, the world. These realms^105^ are hierarchical, nested, and highly interdependent (see Figures 3 and 4) and can be extended to include a geophysical realm, with coevolved geophysical systems providing the foundation for the emergence and subsequent coevolution of life on Earth.^5^

**Figure 4:**
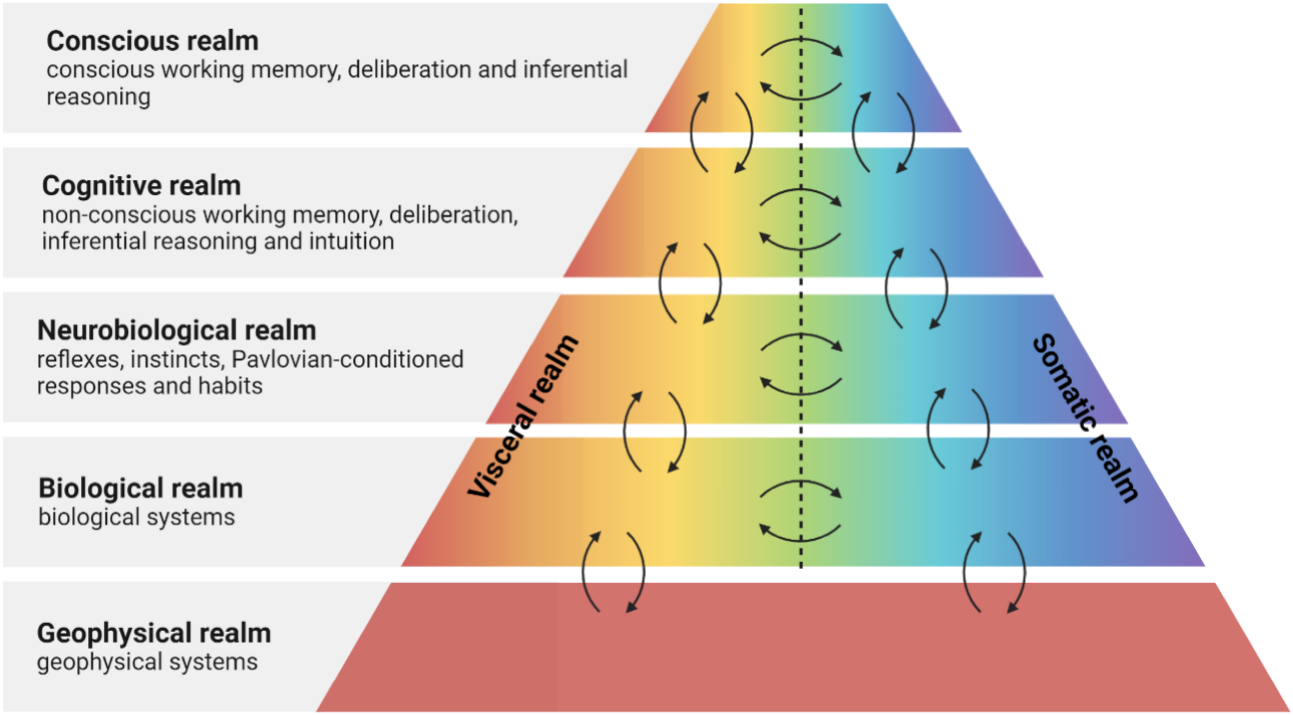
Realms of life on Earth, modified from.^105^ The somatic realm is externally oriented with respect to individual organisms and can be applied at global, regional, urban, and local scales, while the visceral realm is internally oriented with respect to individual organisms and includes organs, cells, organelles, genes, and molecules.

Building on Romer’s conceptualization of the human nervous system,^108^ LeDoux proposed^105^ that interactions of the body with the external world (also referred to as exteroception^109^) are handled by a somatic nervous system, while internal bodily functions (also referred to as interoception^109^) are serviced by a visceral nervous system. This elevates the somatic and visceral nervous systems to a primary level and makes the central and peripheral locations of their neural tissues secondary. From an evolutionary perspective, this makes more sense because the central and peripheral nervous systems are not the targets of evolutionary selection. Instead, the targets were the modular components that performed visceral and somatic functions for the organism.^105^ Indeed, the somatic and visceral realms did not start with animals but exist in all organisms (including plants, fungi, and animals), having begun with our unicellular prokaryotic ancestors and having evolved through unicellular and multicellular eukaryotes,^105^ as shown in Figure 3. This means that the visceral and somatic functions of the primordial biological realm were carried forward into the current biological realm and have also been carried forward into the current neurobiological, cognitive, and conscious realms as animals evolved and diversified.^105^

LeDoux allocates various behavioral control processes^105^ as shown in Figure 4. The neurobiological realm includes noncognitive and nonconscious behavioral control (reflexes, instincts, Pavlovian-conditioned responses, and habits). The cognitive realm includes cognitive but not conscious behavioral control (nonconscious working memory, nonconscious deliberation, nonconscious inferential reasoning, and nonconscious intuition). The conscious realm includes cognitive and conscious behavioral control (conscious working memory, conscious deliberation, and conscious inferential reasoning). Collectively, this amounts to extending the two systems associated with thinking fast and slow^110^ to three systems.^105^ Most importantly, however, the realms encapsulate all life on Earth, including humans. Although there remains considerable debate about current theories of consciousness,^111,112^ LeDoux outlined a theory of consciousness for humans that is consistent with the proposed realms.^105^ Given that the realms include all life on Earth, the potential exists to include consciousness beyond the human case,^113^ although the diagram may need to be revised to recognize cognition in plants and fungi.^78,114^

### 3.3 Anthropocene Systems Are Modular and Hierarchical

Modularity is a focus of research across multiple disciplines, including genetics, developmental biology, functional morphology, population biology, and evolutionary biology^115^ as well as biological, neural, social, linguistic, and electronic networks.^116^ Although modularity is generally recognized as a fundamental feature of all organisms, with profound consequences for evolution,^115^ the concept of modularity clearly depends on the context in which it is used. Our intention is to use evolved modularity to reveal the causally coherent and hierarchical^117–120^ mechanisms that gave rise to our globally connected system of Anthropocene systems.

#### 3.3.1 Phylogenetic Refinement

Given the importance of the neurobiological, cognitive, and conscious realms of life (see Figure 4), and the role these play in facilitating how humans both cause and potentially address societal challenges of the Anthropocene, an essential aspect of the evolutionary perspective is chronicling the morphological and functional modifications to the brain, and the behavioral modifications they enabled.^104,121^ Focusing for now on the evolution of the brain in the human lineage, cumulative additions to adaptive behavior included steering (or taxis navigation) in early bilaterians, reinforcing (or model-free reinforcement learning) in early vertebrates, simulating (or model-based reinforcement learning) in early mammals, mentalizing (involving the use of mental models) in early primates, and speaking (or rhythmic semantic processing) in humans.^104,121^ This theory of phylogenetic refinement^122^ can be used to explain the progressive complexification of brains and the evolved adaptive behavior as the consequence of evolutionary refinement from more basic building blocks. In other words, prior innovations impose constraints on future innovations, meaning that the evolutionary design of biological systems is highly path-dependent. It should be possible to gain similar insights into the form, function, and resulting behavior of the coevolved ensemble of Anthropocene systems, revealing how the realms of life (see Figures 3 and 4) became increasingly complex and interconnected.^5^

#### 3.3.2 Biological and Physiological Circuits

An additional closely related evolutionary insight helps merge our understanding of coevolving Anthropocene systems with biological circuits in systems biology^123^ and physiological circuits in systems medicine,^124^ integrating the deep evolutionary mechanisms that coherently connect all life on Earth.^74^

The archetypal cellular capacities of cooperation, codependence, collaboration, and competition^74,102,103^ began in the primordial biological realm and have been carried forward by coevolution into the current biological, neurobiological, cognitive, and conscious realms.^105^ Similarly, biological and physiological circuits involve networks that can be separated into modular units that perform almost independently.^125^ These network motifs^125–127^ are modular building blocks of the biological circuits of systems biology^123^ and the physiological circuits of systems medicine.^124^ Network motifs, which are also referred to as circuit motifs,^124,128,129^ are basic interaction patterns that recur much more often than in random networks. Network motifs are not randomly distributed in real networks but are combined in ways that maintain autonomy and generate emergent properties.^116^ The same small set of network motifs appears to serve as the building blocks of transcription networks from bacteria to mammals, with specific network motifs also found in signal transduction networks, neural networks, and other biological networks.^123,124^ Each network motif can serve as an elementary circuit with a defined function, including filters, pulse generators, response accelerators, and temporal pattern generators.^123,124^ Evolution appears to have converged on the same motifs, perhaps because they are the simplest and most robust circuits that perform these information-processing functions.^123,124^ These modular building blocks are presumed to have evolved in response to adaptation over evolutionary time scales^130^ resulting in organisms that are highly evolvable and capable of adapting quickly to new goals in coevolving ecological niches.

There is a wide range of biological systems interacting across a range of scales, which are used to process information, make decisions, and achieve specific goals of living organisms.^131^ These modular and hierarchical systems include chemical networks,^132^ neural networks, physiological circuits, individual organisms, and groups of individual organisms within communities.^131^ Evolution has resulted in the progressive selection of existing and novel mechanisms across goal-oriented spaces, enabling adaptive migration toward specific goals in metabolic, physiological, transcriptional, morphological, and behavioral spaces.^133^ Morphological changes involve complex, multiscale feedback mechanisms that influence behavior in ways not directly encoded by genes.^81,133^ Because of this,^134^ we need to move away from considering causes acting at a single site within an organism, instigating changes in a linear pathway, and instead focus on understanding the behavior of the larger interconnected system of systems. Similarly, we need to shift from studying molecular events to studying systemic patterns, which can lead to a shift from medicines that briefly control a single target to treatments that impose constraints on many parts of the organism, sustained over time.^134^ Despite the general awareness of redundancy and homeostatic control circuits, we need a better understanding of the corrective, self-organizing processes that reliably reach complex, systemic goals.^134^

The similarity of network motifs in transcription networks (nanometer-sized molecules interacting on a time scale of hours) and neural networks (micrometer-sized cells interacting on a time scale of less than seconds) is revealing.^123^ While neurons process information between sensory neurons and motor neurons, transcription networks process information between transcription factors that receive signals and genes that act in the inner or outer environment of the cell. This similarity in function suggests that evolution converged on similar network motifs in both networks to perform important information-processing tasks.^123^ Indeed, this evolved modularity is found at all scales of biological organization, including multicellular organisms, organs, unicellular organisms, cells, organelles, genes, and molecules.^123^

The power of this approach is revealed in Alon’s Periodic Table of Diseases.^124^ Using the periodic table as a metaphor, cell types can be classified by both abundance and turnover. This enables a range of diseases (degenerative, progressive fibrotic, autoimmune, toxic adenoma, immune hypersensitivity, and tumor prevalence) to be classified according to organ and cell type.^124^ The resulting table shows six broad patterns aligned with six classes of disease. Most interesting, however, is the fact that each class of disease in the table corresponds to a specific circuit motif.^124^ In addition, the patterns in the table are also relevant from the point of view of age of onset, disease prevalence, and current treatments, as well as suggesting potential future treatments.^124^

#### 3.3.3 Visceral and Somatic Realms

Evolutionary mechanisms gave rise to our system of Anthropocene systems. The resulting globally connected meta-ecosystem has causally coherent mechanisms that span a vast range of scales, starting at the global scale and essentially going “all the way down.” These scales can be identified in different ways, but we can start with global, regional, urban, and local scales, as shown in Figure 1. In addition, the requirement to sustainably balance the health of humans, animals, and ecosystems includes all life on Earth. As shown in Figure 3, living organisms are either unicellular prokaryotes, unicellular eukaryotes, simple multicellular eukaryotes, or complex multicellular eukaryotes. The relevant scales of interest therefore extend down into these living organisms, including organs, cells, organelles, genes, and molecules for complex multicellular eukaryotes; cells, organelles, genes, and molecules for simple multicellular eukaryotes; organelles, genes, and molecules for unicellular eukaryotes; and genes and molecules for unicellular prokaryotes. Encouragingly, the conceptual distinctions between the science of the brain and the body are increasingly being erased, with considerable opportunity for unification into a single conceptual framework.^133^ As previously emphasized, the integrated processing associated with cognition is focused both internally on the visceral realm and externally on the somatic realm (see Figure 4). This provides a useful conceptual boundary to manage the complexity associated with the vast range of scales in our system of Anthropocene systems.

The examples of primary Anthropocene systems we have chosen to identify (summarized in Figure 2) will need to be extended and refined as the evoSoS paradigm is developed, but they can, in principle, be applied across global, regional, urban, and local scales, with individual organisms forming communities and meta-ecosystems. This range of scales is likely the limit for externally oriented conceptual models and an associated computational framework (see Section 4). However, the causally coherent cross-scale mechanisms can be extended down into individual organisms by connecting with evolutionary systems biology and systems medicine, which are already embracing cross-scale systems-oriented frameworks.^123,124,133,135–137^ In this way, internally oriented conceptual models and an associated computational framework could be created, building on current knowledge in evolutionary systems biology, network biology, biomedical engineering, and systems medicine. Interactions between the internally oriented (visceral) and externally oriented (somatic) realms would be orchestrated primarily through the common cognitive system (see Figure 4). As will be emphasized in Section 4, effective communication between these two realms may only be possible if a common language and reconciled ontology are used for both.

## 4 A System-of-Systems Perspective

We have used an evolutionary perspective to outline a theoretical framework that can help us identify and decompose the system of Anthropocene systems, but we still need to create conceptual models and computational frameworks that can help us characterize and converge (or reintegrate) the system of Anthropocene systems. Using a system-of-systems perspective means that we can take advantage of decades of fundamental advances in systems engineering, which has traditionally focused on sociotechnical systems, including human systems integration,^138^ to help address societal challenges of the Anthropocene.^27^ In particular, model-based systems engineering (MBSE),^139,140^ the systems modeling language (SysML)^141^ and hetero-functional graph theory (HFGT)^142,143^ collectively provide a potentially powerful methodology to address these complex challenges. MBSE has evolved as a generic approach to realize a wide range of modeling systems^139^ and is designed to handle systems of substantial scale and complexity. In the following sections, we briefly review conceptual models, modeling languages, ontologies, system architecture, and hetero-functional graph theory from a system-of-systems perspective.

### 4.1 Conceptual Model, Modeling Language, and Ontology

Briefly, a conceptual model^144,145^ of an Anthropocene system of interest (with examples in Figure 2) has a purpose, a boundary, and system elements that interact with one another across well-defined interfaces, creating system form and function. The boundary defines the scope of the system and can be either physical or conceptual. The system and the elements have well-defined attributes, requirements, and constraints. The attributes include functions, which, together with the system form, create the behavior of the system. Stakeholders have an interest in the system but are outside the boundary of the system of interest. There may be other enabling systems that also lie outside the boundary of the system of interest, interacting with the system of interest through well-defined interfaces at the system boundary. Systems that are hierarchical are also possible, where system elements can be aggregated (zooming out) or disaggregated (zooming in). Finally, a system of systems can be created in which the system elements of the system of interest are themselves systems.

The need for a system-of-systems perspective arises because of problems with complexity (e.g., when complexity is not identified and therefore cannot be managed or controlled), communication (e.g., when communication fails or is ambiguous), and understanding (e.g., when different points of view are not taken into account and incorrect assumptions are made), with the three problems collectively compounding one another.^139^ When developing a model of an Anthropocene system, one of the main approaches to improve communication (which occurs among the people, organizations, and stakeholders who develop and use a model, as well as between and within systems and system elements) is to use a common language.^139^ In fact, MBSE uses a combined spoken and visual common language (the systems modeling language, or SysML) as well as multiple domain- or discipline-specific languages, all of which need to be managed as effectively as possible. For example, SysML can be thought of as a dialect of the unified modeling language^139^ which was created to manage communication in complex software systems. As emphasized in Figure 5, the combined semantic and graphical nature of SysML is very useful when attempting to simultaneously improve communication and reconcile the vast array of domain- or discipline-specific ontologies in a system of Anthropocene systems.

**Figure 5:**
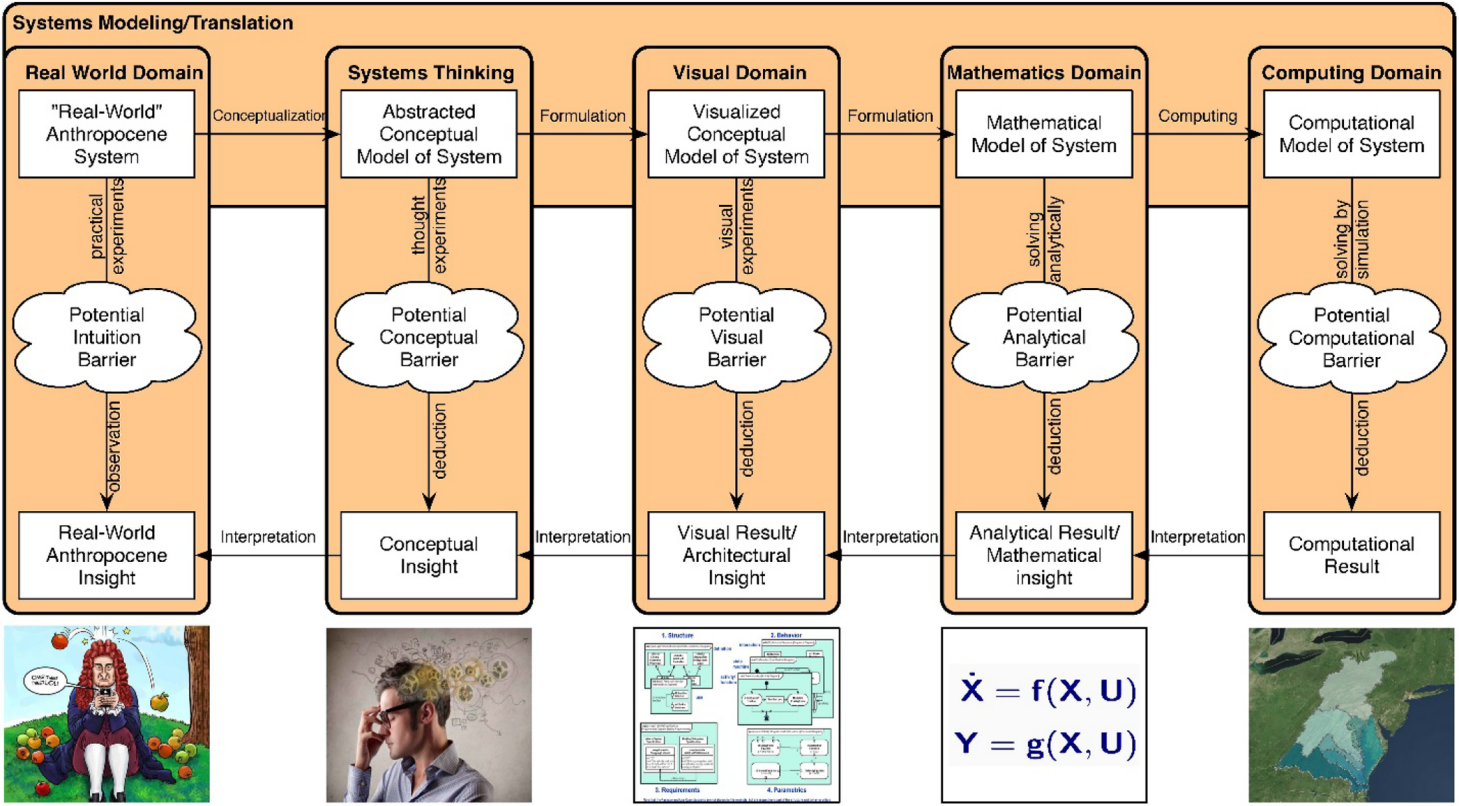
Developing models of Anthropocene systems involves the creation and use of scientific knowledge and the subsequent translation of this knowledge among the real world, systems thinking, visual, mathematical, and computing domains.

From a systems engineering perspective, an ontology can be thought of as a formal, explicit specification of a shared domain conceptualization^146^ describing the relationship between reality (the knowledge domain), the understanding of reality (the domain conceptualization), and the description of reality (using a language). Ontologies can make the form and function of systems and their elements explicit and can help stakeholders better understand the complexities inherent in large systems.^146^ A conceptual model^145^ of a system of systems needs a well-defined foundational, universal, general, necessary, and sufficient ontology that renders concepts and terms precise and unambiguous.^146^ Ontologies avoid ambiguity and provide an accepted and consistent vocabulary, facilitating semantic interoperability among humans as well as between humans and computers.^146^

Now consider the convergence challenge associated with a system of heterogeneous Anthropocene systems,^142^ each with its own domain conceptualization and associated language and ontology. First, humans are typically trained in a single domain conceptualization rather than multiple domain conceptualizations. Indeed, it is doubtful that a single human—or even many—has sufficient knowledge of multiple domains. A group of individuals, each with their own individual domain conceptualization, must therefore collaborate and reach agreement on the integration of multiple domain conceptualizations. They immediately find that each domain conceptualization comes with its associated language, and a language of languages emerges. Because each of these languages was developed independently to address the needs of its associated domain, a common, convergent understanding among languages is difficult to achieve. It is possible that the language of languages develops a translation capability between each of the languages for each domain, but this does not scale when there are N domains that require N­(N – 1) translators between N languages (e.g., 90 translators are needed for 10 systems). The only alternative is to invest in the development of a language that reconciles the individual languages into a single common language. For these reasons, HFGT adopts a single common language (SysML) that serves as a language of languages. For a system of systems, this requires instantiated, reference, and meta-architectures.

### 4.2 System Architecture

System architecture generally consists of three parts: the structural architecture (i.e., form), the functional architecture (i.e., function), and the mapping of function onto form in a system concept or allocated architecture. The structural architecture is a description of the decomposed elements of the system, without any specification of the performance characteristics of the system resources that comprise each element. The functional architecture is a description of the system processes in a solution-neutral way, structured in serial or parallel, and potentially in hierarchical arrangements. The system concept, which is a mapping of the functional architecture onto the structural architecture, completes the system architecture.

An instantiated system architecture is a case-specific architecture that represents a real-world scenario. At this level, the structural architecture consists of a set of instantiated system resources, and the functional architecture consists of a set of instantiated system processes. The mapping in the system concept defines which resources perform which processes.

The reference architecture generalizes instantiated system architectures. Instead of using individual instances as elements of the structural and functional architecture, the reference architecture is expressed in terms of domain-specific classes of these instances. In this way, the reference architecture captures the essence of existing instantiated architectures. It also provides a vision of future needs that can provide guidance for developing new instantiated system architectures. Such a reference architecture facilitates a shared understanding across multiple disciplines or organizations of the current architecture and its future evolution. A reference architecture is based on concepts proven in practice. Most often, preceding architectures are mined for these proven concepts. The reference architecture, therefore, generalizes instantiated system architectures to define an architecture that is generally applicable in a discipline or knowledge domain. However, the reference architecture does not generalize beyond domain conceptualization.

The meta-architecture further generalizes reference architectures. Instead of domain-specific elements, it is expressed in terms of domain-neutral classes. A reference architecture is composed of primitive elements that generalize the domain-specific functional and structural elements into domain-neutral equivalents. While no single engineering system meta-architecture has been developed for all purposes, several modeling methodologies have been developed that span several discipline-specific domains. In the design of dynamic systems, bond graphs^147^ and linear graphs^148^ use generalized capacitors, resistors, inductors, gyrators, and transformers as primitive elements. In system dynamics, stocks and flows are used as primitives,^149^ while in graph theory^150,151^ nodes and edges are used as primitive elements. Each of these domains has its own set of applications. However, their sufficiency must ultimately be tested by an ontological analysis of soundness, completeness, lucidity, and laconicity (for more detail, see ref. 142). Hetero-functional graph theory utilizes its own meta-architecture that has been shown to generalize linear graphs, bond graphs, formal graph theory, system dynamics, hydrologic systems, and economic input-output systems.^143,152–156^ Given the importance of ontological clarity, HFGT takes special care in the translation of this meta-architecture from its description in SysML^141^ to its mathematical and computational representations, as shown in Figure 5.

### 4.3 Hetero-Functional Graph Theory

HFGT^142,143^ is a fusion of network science (including formal graph theory and multilayer networks) and MBSE. Graph theory focuses primarily on an abstract model of a system’s form, neglecting an explicit description of a system’s function. For example, in a formal graph with nodes and edges, nodes typically represent locations, while edges represent connections between nodes. The nodes and edges in a formal graph are described by nouns. Because many complex systems include multiple elements with several layers of connectivity, formal graphs are frequently scaled up to create multilayer networks (e.g., ref. 157). In either case, operands (which include matter, energy, information, and individual organisms) can be used to connect the edges and the nodes. In real-world Anthropocene systems, however, operands are subject to both transport and transformation processes as they move between nodes. HFGT overcomes the limitations of formal graphs and multilayer networks (for example, it has been shown that HFGT overcomes eight previously identified modeling constraints in multilayer networks^142,158^ ), enabling the inclusion of nouns and verb phrases that are needed to describe system form and function.

Figure 6 represents the meta-architecture for HFGT expressed in SysML. A reference architecture describes all the potential system capabilities, while an instantiated version of this reference architecture includes multiple operands, capabilities, buffers, system resources, and system processes. As shown in Figure 6, HFGT makes the connection to the common language explicit through a set of system resources as subjects, a set of system processes as predicates, and a set of operands as their constituent objects. In this way, system processes can be allocated to system resources to create subject + verb + object sentences called system capabilities. As a result, SysML and HFGT together create a common language and computational framework, providing the means to produce an ontologically coherent computational model. The abstract nature of the meta-architecture is highly extensible, meaning that new operands, new resources, and new processes can be added as required. In addition, HFGT is highly scalable, meaning that elements of the reference architecture can be instantiated as many times as needed.

**Figure 6:**
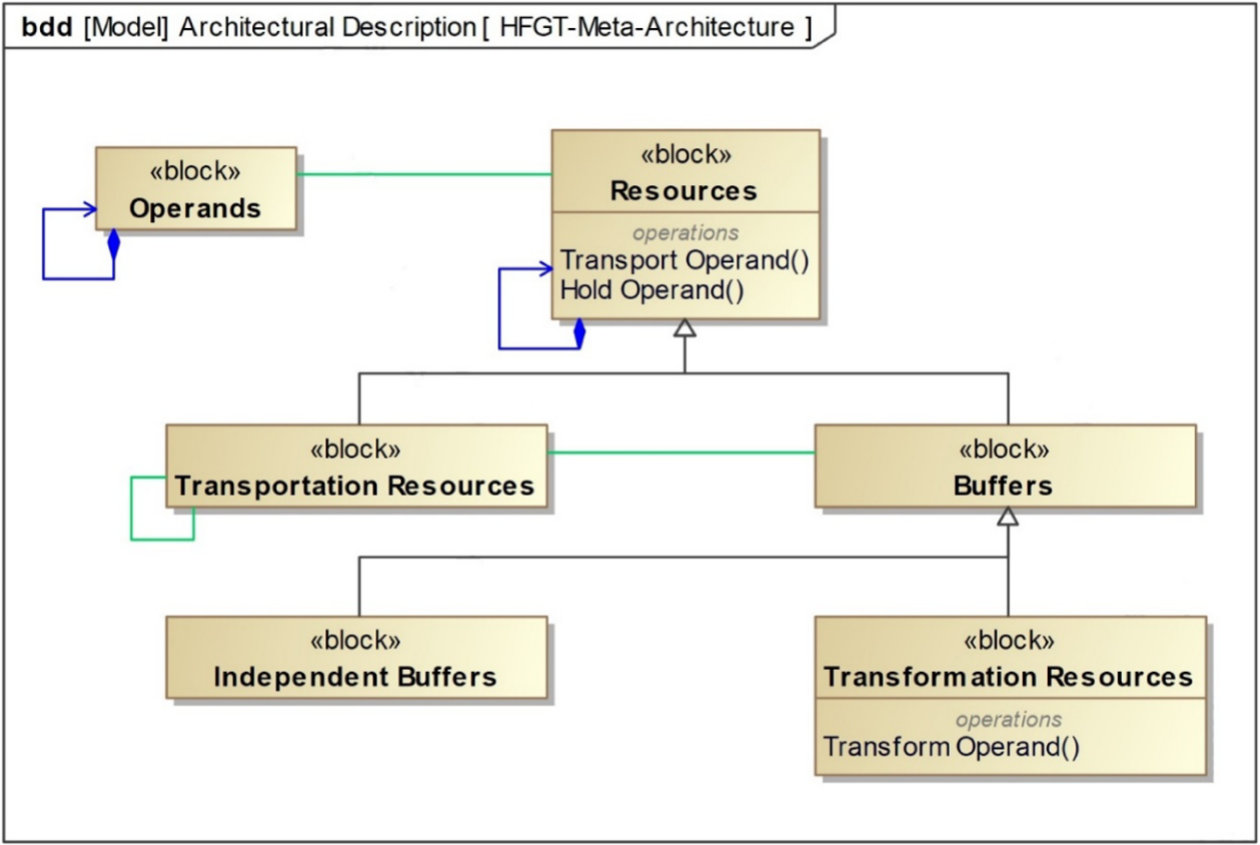
Hetero-functional graph theory (HFGT) meta-architecture^142^ represented using the systems modeling language (SysML). The HFGT meta-architecture consists of three types of resources (transportation resources, independent buffers, and transformation resources) that are capable of two types of processes (transport operand, which implicitly includes hold operand, and transform operand). Lines between the blocks indicate various associations that define structural relationships and visually represent how system elements are connected or composed.^141^

HFGT can be used to conduct analyses of system form as well as simulations of system behavior.^142,158–165^ HFGT has already demonstrated its relevance to convergent Anthropocene systems, with results in electric power, water distribution, natural gas, oil, coal, hydrogen, transportation, manufacturing, and healthcare systems.^142,160–165^ Perhaps more importantly, it has been used for combinations of these systems, such as the American Multi-Modal Energy System,^166^ which is a system of systems comprised of four separate but interdependent infrastructure enterprises. HFGT can model an arbitrary number of systems of arbitrary size and topology connected to each other in an arbitrary manner.^142^ In essence, HFGT begins with a generic meta-architecture (Figure 6) that is independent of any system and then uses this to create a computational model of a specific system, which is hierarchical, extensible, and scalable.

## 5 An Evolutionary, System-of-Systems, Convergence Paradigm

To simultaneously address One Earth and One Health, the evoSoS convergence paradigm requires that evolutionary scientists, behavioral scientists, natural scientists, health scientists, systems scientists, and engineers systematically identify, decompose, characterize, and then converge the nested, evolutionary ensemble of geophysical, biophysical, sociocultural, and sociotechnical systems. Here, we briefly describe the five primary elements of the paradigm (theoretical framework, conceptual models, computational frameworks, decision-support system, and educational pedagogy), as shown in Figure 7.

**Figure 7:**
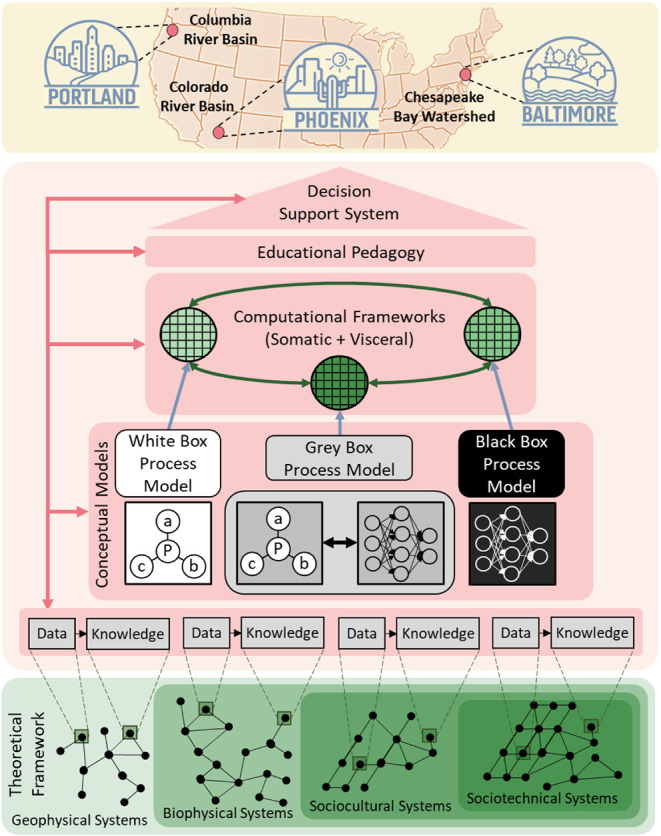
Schematic representation of an agile, evolutionary, system-of-systems convergence paradigm for holistically framing and addressing One Earth, One Health, and the associated interdependent societal challenges of the Anthropocene.

### 5.1 Theoretical Framework

The first primary element is a causally coherent, scientifically falsifiable theoretical framework characterizing a system of Anthropocene systems within a planetary-scale meta-ecosystem. The framework includes individual organisms engaging in niche construction in a globally connected meta-ecosystem. Living organisms that can be represented include unicellular prokaryotes, unicellular eukaryotes, simple multicellular eukaryotes, and complex multicellular eukaryotes, essentially spanning all life on Earth, including plants, fungi, and animals (Figure 3). The framework is based on the geophysical, biological, neurobiological, cognitive, and conscious realms of life, integrating the deep evolutionary mechanisms of all Anthropocene systems. The causally coherent, cross-scale mechanisms can be applied at global, regional, urban, and local scales in the somatic realm but can also be extended down into individual organisms at scales that include organs, cells, organelles, genes, and molecules in the visceral realm (Figure 4).

A theoretical evolutionary framework, founded on an understanding of how Anthropocene systems coevolved and became increasingly interconnected, is crucially important when relating Anthropocene systems to human cognition, communication, and the resulting human behavior.^92^ This is because it was the evolution of the human brain,^121,167^ combined with the evolution of culture and technology, that drove the evolution of the Anthropocene. The way we think, the way we communicate, and the way we make decisions and transform decisions into behavior all influence, and are influenced by, the coevolving geophysical, biophysical, sociocultural, and sociotechnical systems in which our lives are entwined.^5^ An Earth-system-based approach that holistically accounts for system-wide human–environment interactions^3^ must ultimately include all Anthropocene systems, from the global scale down to molecules in individual organisms.^4^

### 5.2 Conceptual Models

The second primary element comprises cross-scale, modular, hierarchical, dynamic, and conceptual models of the Anthropocene systems that are based on a common language and that reconcile disciplinary ontologies. The theoretical framework can be translated into conceptual models that characterize the form and function of the entire system of Anthropocene systems. Developing causally coherent models with well-established mechanisms is the most reliable way^168–170^ to improve our understanding of meta-ecosystems that span multiple scales. The models should also be hierarchically coherent, but this is facilitated by the evolved nature of Anthropocene systems (e.g., see refs. 171,172 and Figure 3). As shown in Figure 7, the evoSoS convergence paradigm can accommodate mechanistic “white box” models, theory-guided, machine learning “grey box” models, as well as machine learning “black box” models, with the potential to move from black box to grey box to white box models as mechanistic understanding is gained (e.g., see ref. 155). Although SysML, which was designed for sociotechnical systems including human systems integration,^138^ is proposed as the common visual and spoken language for the conceptual models, it may need to be retooled for some Anthropocene systems.^27^ For example, integrating SysML with existing standards such as SBML (the systems biology markup language)^173^ may be of value.

### 5.3 Computational Frameworks

The third primary element comprises common computational frameworks that build directly on the conceptual models and that are agile, extensible, and scalable. We currently envision two interoperable computational frameworks (one for the somatic realm and one for the visceral realm, as described in Section 3) that can be applied at the relevant scales of interest within each realm. SysML is used to create ontologically coherent conceptual models using common spoken and visual language, while HFGT builds directly on the conceptual models, providing the means to produce ontologically coherent computational models. Within the conceptual models and associated computational frameworks, the operands that are subject to transport and transformation processes can include matter, energy, information, and individual organisms. As a result, we can, in principle, develop models of an ensemble of geophysical, biophysical, sociocultural, and sociotechnical systems that include niche construction in meta-ecosystems and that can be instantiated at the various scales of interest in the somatic and visceral realms.

MBSE,^27^ SysML,^141^ and HFGT^142^ provide a potentially powerful way to frame and address complex societal challenges. As shown in Figure 5, the methodology first translates real-world Anthropocene systems into SysML to integrate and reconcile ontologies^141^ and then uses HFGT^142,143^ to algorithmically traverse the gap from the graphical SysML model to the associated mathematical model, and ultimately to the computational model. HFGT is especially helpful, as it can be used to coherently span spatial and temporal scales. Models that are based on mass and energy balances (which is often the case for geophysical systems, and the technical subsystems of sociotechnical systems) are well-suited for spanning spatial scales with HFGT.^155^

### 5.4 Decision-Support System

The fourth primary element is a coherent decision-support system used to interact with the conceptual models and computational frameworks, enabling effective integration of a wide range of stakeholder perspectives spanning multiple scales and organizational levels.

Developers of decision-support systems face stakeholder-oriented, model-oriented, and system-oriented issues, with a recent review^174^ providing recommendations on how to build them. Approaches include stakeholder engagement and participatory modeling, constructing future scenarios while balancing synergies and trade-offs across multiple systems, and supporting decision-making under deep uncertainty.^41,175,176^ An evoSoS decision-support system must provide salient insights about interventions and scenarios in a manner that aligns with stakeholder affect and cognition.^177^ When possible, computational results should be visualized to support graphical storytelling so that real-world insights are gained easily and decisions are made effectively.

Recent work on strategic environmental crisis management offers guidelines for the design of decision-support systems capable of integrating knowledge on issues of high complexity and uncertainty. The challenge is to address long-term path dependencies while navigating urgent anthropogenic crises^177^ and decision-support systems should provide a platform for egalitarian deliberations among experts and policymakers. The agenda for the deliberations should be structured around alternative futures that provoke the imagination and facilitate the critical questioning of cognitive biases. Tools to enhance imagination and questioning include audio-visual dashboards that take the decision-makers to an imagined future, illustrate the implications of the decisions considered,^178^ and facilitate analysis of how strategic interventions can fail under plausible disruption scenarios.

Societal challenges involving Anthropocene systems are characterized by deep uncertainty, with many approaches to decision-making that enable quantitative analyses and support deliberation among multiple parties.^179^ These methods can be used to generally identify robust or low-regret management strategies that perform well across a wide range of uncertain conditions. From a holistic perspective, the goal should be to optimally manage both complexity and uncertainty.

### 5.5 Educational Pedagogy

The fifth primary element is a comprehensive educational pedagogy designed to train a new generation^24^ of Anthropocene System Integrators (including students, academics, practitioners, and stakeholders) to develop and implement the paradigm. We envision at least seven components to the evoSoS pedagogy: (1) an introduction to the theoretical evolutionary framework, including an overview of our “origin story” which reveals the nested ensemble of geophysical, biophysical, sociocultural, and sociotechnical systems;^5^ (2) a clear understanding of the causally coherent, cross-scale, conceptual models of a system of Anthropocene systems; (3) convergent Anthropocene-systems thinking as a translation from real-world systems to SysML; (4) HFGT as a translation from SysML to mathematical and computational models; (5) data analytics, visualization, and machine learning; (6) stakeholder-based decision-support systems; and (7) principles of convergence,^30^ team science^180^ and good modeling practice.^181–183^

## 6 Developing and Implementing the SoS and evoSoS Convergence Paradigms

The system-of-systems convergence paradigm^184^ is being developed and implemented with support from a National Science Foundation Growing Convergence Research project that focuses on three interdependent societal challenges (agricultural impacts in the watershed, eutrophication of the estuary, and regional economic growth) in the Chesapeake Bay Watershed region (see Figure 7), focusing initially on three interdependent systems (land-use, watershed, and estuary). After expressing land-use and watershed models in SysML, we are integrating them using HFGT.^154,155^ Unified continuity and constitutive laws are applied across multiple model elements, generating an extensible and scalable simulation structure that integrates land-use segments, outlet points, river segments, and the estuary.^154,155^ We are adding an economic system^156^ and can include other relevant systems as needed (e.g., see ref. 153). Our decision-support system is based on SysML^185^ and will draw on the HFGT computational framework to simulate scenarios of interest and perceived trade-offs. We are developing our educational pedagogy^186^ to train a new generation of Anthropocene systems integrators, using SysML as the common language and HFGT as the common computational framework.

Although the SoS convergence paradigm has been initiated, further research on the theoretical evolutionary framework is required, as described in Section 7. The combined evoSoS convergence paradigm^5,187^ could then be tested by building on the SoS convergence paradigm (i.e., Chesapeake Bay Watershed + Baltimore, as shown in Figure 7), with the potential to examine causally coherent strategic interventions across regional, urban, and local scales by zooming into Baltimore. The approach could then be extended to other regions with associated urban areas (e.g., Colorado River Basin + Phoenix and Columbia River Basin + Portland, as shown in Figure 7) illustrating how the evoSoS convergence paradigm can be used to facilitate the required communication, coordination, capacity building, and collaboration, and eventually to the global scale with strategically selected regions around the world.

## 7 Research Needs

Humans have been addressing societal challenges since our species evolved roughly 200,000 years ago. An important difference now is that we are using scientific research to help us address societal challenges that are far more complex than those previously attempted. While this is an exciting opportunity for research, the fragmented nature of the prevailing academic and scientific culture^17^ is arguably the biggest barrier that prevents us from using our rapidly accumulating collective knowledge more effectively.

Holistically addressing One Earth and One Health requires communication, coordination, capacity building, and collaboration.^6^ However, these crucial requirements will be essentially impossible to achieve without a common language and reconciled ontology, common conceptual models, and common computational frameworks. The proposed evoSoS convergence paradigm attempts to address these requirements. Although we again acknowledge the daunting and ambitious nature of the paradigm,^5^ and again emphasize that we seek neither to model everything nor to predict the future,^5^ holistically addressing the family of societal challenges can only begin with a broad overview of the entire knowledge domain, including all Anthropocene systems.

The evoSoS convergence paradigm intends to address One Earth and One Health in a holistic fashion, requiring coordinated interventions across multiple systems and scales. However, as we change scale from global to regional to urban to local in the somatic realm and move from organs to cells to organelles to genes to molecules in the visceral realm, it should be clear that potential interventions are scale-dependent, with different intervention opportunities and transformation pathways becoming accessible as we zoom in or out. We therefore need a causally coherent meta-ecosystem model that applies over the range of scales of interest. The model should also be hierarchically coherent, which is inherently facilitated by the evolved form and function of the Anthropocene systems. Unfortunately, we are not aware of any theoretical frameworks or conceptual models where coevolved systems are identified and decomposed from the larger system of Anthropocene systems and then coherently characterized in a way that will enable their convergence, clarifying the cross-scale causal connections among the various systems. Although the range of scales might ultimately be defined differently, starting with these specific scales means that individual organisms would be represented at the local scale, with One Earth focusing more on the somatic realm and One Health focusing more on the visceral realm, but remaining closely integrated, as shown in Figure 4.

A major coordinated initiative is needed to develop cross-scale models of sociocultural systems and their causally coherent connections with other Anthropocene systems. While all causal influences are clearly not equally important, human behavior influences and is influenced by the globally connected system of Anthropocene systems. An outline of a more generic model of a sociocultural system is given in Section 3.1, with social dynamics that involve individuals, groups, and groups of groups, providing a way to scale these interacting systems coherently. Indeed, there is growing recognition that a complex systems approach is required to represent the multiscale, multidimensional, dynamic, and interacting nature of sociocultural systems (e.g., see refs. 188,189 ). To be successful, however, we must overcome the fragmented nature of research on human behavior (e.g., see ref. 63) enabling a more coherent integration of sociocultural systems and their causally coherent connections with other Anthropocene systems (see Figure 2). Furthermore, the need to sustainably balance the health of humans, animals, and ecosystems means that we must overcome the fragmented nature of research on human, animal, and ecosystem behavior, enabling a more coherent integration across the realms of life (see Figure 4).

Fortuitously, network biology^136^ is currently focused on gaining a comprehensive understanding of the entire cellular or organismal interactome across different conditions and life stages, including maps of every biological interaction in an organism, from molecules and genes up to tissues and organs. In addition, the integration of network biology with other disciplines provides a holistic understanding of life, connecting the molecular interactome with tissue-level networks, organ systems, as well as interorganismal interactions, such as those in ecosystems.^136^ Furthermore, machine learning provides a powerful tool for creating biological models with tunable parameters that operate on structured data, with recent methods designed to produce graph elements (e.g., nodes, edges, subgraphs, and entire graphs) that capture essential information about the topology of these elements.^136^ These developments in network biology are promising because our proposed evoSoS convergence paradigm overcomes many of the limitations of multilayer networks (e.g., see refs. 190–192 ), as emphasized in Section 4.

The required communication, coordination, capacity building, and collaboration will be facilitated by conceptual models, which are cross-scale, modular, and hierarchical, as well as computational frameworks, which are agile, extensible, and scalable. However, we need new research programs that can facilitate this much broader research agenda. For example, national and global funding agencies could solicit research on the best approaches to identify, decompose, characterize, and then converge the system of Anthropocene systems. This would enable the scientific evaluation of similar competing convergence paradigms but could also enable the emergence of a global community of practice (e.g., ref. 193) to develop community models (e.g., refs. 193 and 194,195 ) for specific Anthropocene systems that can be integrated within a wide range of geophysical, biophysical, sociocultural, and sociotechnical contexts. SysML can be used to create reference architectures for multiple Anthropocene systems that are shared on open-science platforms (e.g., the Open Modeling Foundation^196,197^ ) and ultimately linked to a cloud-based computational environment (e.g., the HFGT Toolbox^159^). Effective capacity building will also require an agile approach,^27,198^ meaning that the development and implementation of the evoSoS convergence paradigm should take place in carefully planned iterations. We too often invest in incremental approaches because they offer short-term insight, without asking whether they lead to analytical dead ends.

The 50-year-old saying^80^ that “nothing in biology makes sense except in the light of evolution” has recently been extended to both cultural evolution^199^ and cognition-based evolution.^81,82,112^ Although it now appears that nothing in the Anthropocene makes sense except in the light of geological, genetic, cultural, and technological evolution, it is humbling to recall that 100 years ago Smuts^200^ proposed: “Holism... is the principle which makes for the origin and progress of wholes in the universe” and “Evolution is nothing but the gradual development and stratification of a progressive series of wholes, stretching from the inorganic beginnings to the highest levels of spiritual creation.”

## Data Availability

There are no new data sets associated with this manuscript.
